# Multidrug-Resistant *Klebsiella pneumoniae* ST307 in Traveler Returning from Puerto Rico to Dominican Republic 

**DOI:** 10.3201/eid2508.171730

**Published:** 2019-08

**Authors:** Rita Rojas, Nenad Macesic, Gilda Tolari, Anel Guzman, Anne-Catrin Uhlemann

**Affiliations:** Hospital General Plaza de la Salud, Santo Domingo, Dominican Republic (R. Rojas, G. Tolari, A. Guzman);; Monash University, Melbourne, Victoria, Australia (N. Macesic);; Columbia University Medical Center, New York, New York, USA (N. Macesic, A.-C. Uhlemann)

**Keywords:** Carbapenem-resistant Enterobacteriaecae, MDR, Klebsiella pneumoniae, multidrug resistance, Dominican Republic, Puerto Rico, bacteria, United States

## Abstract

We report *bla*_KPC-2_–harboring carbapenem-resistant *Klebsiella pneumoniae* in an emerging sequence type 307 lineage in a traveler returning from Puerto Rico to the Dominican Republic. Phylogenetic analyses indicate regional dissemination of this highly drug-resistant clone across the Americas, underscoring the need for adequate surveillance and infection control efforts to prevent further spread.

Carbapenemase-resistant *Enterobacteriaceae* (CRE), in particular carbapenem-resistant *Klebsiella pneumoniae* (CRKp), represent a serious threat to public health (*1*). CRKp infections have been associated with high mortality rates, up to 50% in some studies (*2*). In resource-limited regions, such as the Dominican Republic, multiple challenges hinder efforts to contain CRE infections, including lack of novel antimicrobial drugs, inability to monitor drug levels of potentially toxic treatment regimens, and absence of molecular tools to investigate outbreaks and potential spread.

In fall 2015, a 66-year-old woman with diabetes mellitus, hepatitis C virus infection, and end-stage renal disease on hemodialysis was admitted to a hospital in the Dominican Republic for fever, anorexia, chills, and myalgia. On day 3, her blood culture tested positive for *K. pneumoniae.* She had been admitted to a hospital in Puerto Rico a few months before and had been treated for a multidrug-resistant bacterial infection.

The *K. pneumoniae* isolate from the patient was nonsusceptible to all tested antimicrobial drugs except polymyxins (Appendix Table 1). We began combination therapy with a loading dose of colistin, then 100 mg postdialysis, plus ertapenem (150 mg postdialysis) and fosfomycin (2 g 3×/d). We implemented infection control measures by placing the patient in a single room and using gloves, gowns, masks, and a dedicated stethoscope. Despite initial improvement, the patient died on day 25 after admission.

Whole-genome sequencing revealed that the patient isolate, NR6025, was of the emerging sequence type 307 (ST307) (*3*) and closely related (<185 SNPs) to several international ST307 isolates of similar phenotype (Figure). Of note, this isolate was most closely related, within 36 SNPs, to an isolate recovered from a patient in New York, NY, USA, who also had been hospitalized in Puerto Rico in 2016 (*4*). This finding raises the possibility that both patients acquired CRE in Puerto Rico and their infections subsequently developed in their home countries.

**Figure:**
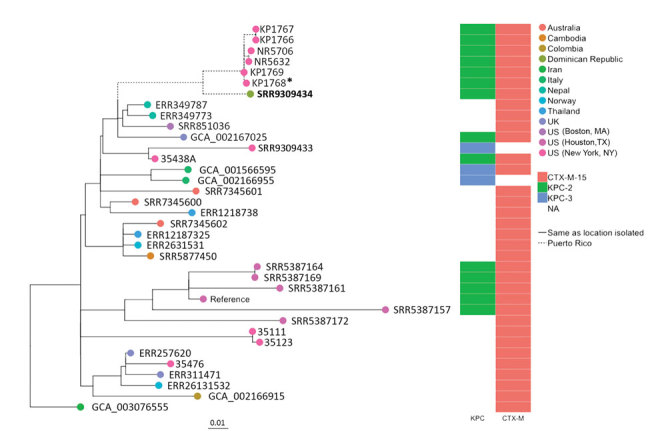
Maximum-likelihood phylogenetic tree of geographically diverse *Klebsiella pneumoniae* sequence type 307 isolates based on 860 concatenated single-nucleotide polymorphisms, extracted from an alignment length of 5,248,133 bp. Bold indicates isolate from a traveler from Puerto Rico to the Dominican Republic (this study). Asterisk (*) indicates an isolate recovered from a patient admitted to a hospital in Puerto Rico during the same year as the case-patient in this study (*4*). *bla* gene types (KPC, CTX-M) are indicated. Scale bar indicates nucleotide substitutions per site. NA, not applicable.

In silico resistance gene detection demonstrated that *bla*_KPC-2_, on Tn*4401e*, was likely the mechanism of carbapenem resistance for this isolate. Moreover, the meropenem MIC was >32 μg/mL, consistent with high carbapenem MICs observed in the ST307 Tn*4401e* isolates (*4*) from New York, suggesting association with a strong promoter. In addition, the isolate harbored a large repertoire of acquired-resistance genes, including additional β-lactamase genes CTX-M-15, SHV-100, OXA-1, and TEM-1D (Appendix Table 1). The isolate contained IncFIBK, ColRNA1, and IncA/C2 plasmid replicons; IncA/C plasmid encodes for *bla*_KPC-2_, *bla*_TEM_, *sulI*, *aadB*, *aac6*, and *qacE*, which has been implicated in chlorhexidine resistance.

A case of CRKp was described from Medellin, Colombia, in 2005, and subsequent CRKp infections have been reported in Mexico, in South America in Brazil, Argentina, and Venezuela, and in the Caribbean in Cuba, Puerto Rico, and Trinidad and Tobago (*5*–*7*). In many of these studies, CRKp isolates were mainly accounted for by ST258 and ST512. The SENTRY Antimicrobial Surveillance Program showed that *bla*_KPC-2_-harboring CRE accounted for most CRE infections in Latin America and that the incidence rate has been rising sharply (*8*). These organisms also are prevalent in Puerto Rico, where a 6-month, PCR-based, islandwide hospital surveillance study conducted in 2011 found that 333/2,805 (11.9%) *K. pneumoniae* isolates harbored *bla*_KPC_ (*9*). However, little is known about CRKp genotypes in Puerto Rico.

Our case highlights the many challenges for controlling CRE infections in resource-limited countries like the Dominican Republic and accentuates the potential for international spread of CRKp through travel, particularly between resource-limited regions. Rapid molecular diagnostic tests for CRKp are not widely available, which can delay treatment. Optimal treatment regimens for CRKp remain controversial, but combination therapy could reduce risk for death compared with monotherapy (*10*). Our facility lacked the resources needed to monitor colistin drug levels, a major concern in particular in patients with underlying renal dysfunction. 

Risk factors for acquisition of CRE in resource-limited settings are not well defined, potentially delaying diagnosis and implementation of infection control strategies. In our case, recent travel, healthcare contact, and unspecified exposure to antimicrobial drugs might have played a role in the patient’s CRE infection. We did not observe additional CRKp infections at our institution during a 6-month follow-up period after this case. However, we were unable to institute an active molecular surveillance program. We cannot rule out silent transmission and colonization of other hospitalized patients or contacts. Even though 2 cases have now been linked to travel to Puerto Rico, no molecular epidemiologic data are available from that island. Future studies should target active surveillance for CRKp in the Caribbean.

Of note, although ST258 has been the dominant genotype of the CRE epidemic globally, the ST307 clone could be expanding disproportionately in some locations. For example, in Houston, Texas, USA, ST307 now accounts for more *K. pneumoniae* infections than ST258 (*3*). Moreover, ST307 Tn*4401e* *bla*_KPC-2_ isolates showed high carbapenem MICs. Taken together, our data suggest that ST307 is highly drug resistant and harbors an extended repertoire of antimicrobial resistance genes, which might have accelerated its recent emergence and wide dissemination.

Appendix Additional information on multidrug-resistant *Klebsiella pneumoniae* due to *bla*_KPC-2_ ST307 in a patient in the Dominican Republic with recent travel to Puerto Rico.
