# Supplementary material for: Multidrug-Resistant Klebsiella pneumoniae ST307 in Traveler Returning from Puerto Rico to Dominican Republic
Source: Emerg Infect Dis. 2019 Aug;25(8):1583–5. doi: 10.3201/eid2508.171730 (PMC6649323; doi:10.3201/eid2508.171730)
Supplement: Appendix — Additional information on multidrug-resistant Klebsiella pneumoniae due to blaKPC-2 ST307 in a patient in the Dominican Republic with recent travel to Puerto Rico. [file 17-1730-Techapp-s1.pdf]

Article DOI: https://doi.org/10.3201/eid2508.171730

Multidrug-Resistant *Klebsiella pneumoniae* ST307 in Traveler from Puerto Rico to Dominican Republic

Appendix

Detailed Clinical Case

The patient and family did not recall the source of the infection in Puerto Rico or treatment regimen, but endorsed that she had experienced adverse reactions to her antimicrobial treatments. Further workup, including transthoracic echocardiogram, revealed a vegetation of 0.4 X 0.5 cm on the tip of the hemodialysis catheter on the right side of the heart, which was subsequently removed. The patient remained bacteremic for 6 days after initiation of treatment. She clinically improved and her procalcitonin levels decreased from ≥200 ng/mL to 29 ng/mL. We did not have the ability to measure colistin levels in our laboratory. However, on day 11 of treatment, the patient began to experience neuropathy and diarrhea and fosfomycin was discontinued. On day 25 of admission the patient experienced cardiac arrest and died.

Antimicrobial Susceptibility Testing

We performed antimicrobial susceptibility testing by using Vitek2 Compact (bioMérieux, https://www.biomerieux.com) and interpreted susceptibilities according to Clinical and Laboratory Standards Institute (CLSI) guidelines (*1*). The patient's isolate was nonsusceptible to all tested antimicrobial drugs, except polymyxin (Table 1). Fosfomycin susceptibility testing and broth microdilution testing for polymyxin was not initially available at our institution and was performed after the patient died according to CLSI guidelines (*1*). Modified Hodge's test was positive, indicating presence of a carbapenemase.

Whole Genome Sequencing and Bioinformatics Analyses

We extracted DNA from bacteria cultured overnight by using the UltraClean Microbial DNA Isolation Kit (QIAGEN, https://www.qiagen.com). We prepared libraries by using Nextera XT DNA Library Prep Kit and sequenced on MiSeq (Illumina, https://www.illumina.com).

We performed SRST2 analysis (*2*) for multilocus sequence typing and characterization of resistance determinants. For comparative sequence analyses, we mapped Illumina reads against a *K. pneumoniae* ST307 reference genome (GenBank accession no. GCA_002148835.1) and included additional, previously published sequences for comparative analyses (Appendix Table 2) (*3*–*5*). We performed variant calling by using Snippy 3.1 after exclusion of mobile genetic elements with PHASTER and IslandViewer 3 (*6*–*8*).

For phylogenetic analyses, we generated a core chromosomal single nucleotide variant alignment by using Snippy 3.1 (*6*). We used a maximum likelihood approach with RAXML to construct a phylogenetic tree based on 860 concatenated SNPs (*9*). We assessed support for nodes by using 1,000 rapid bootstrap inferences and then by a thorough maximum likelihood search. We estimated free model parameters by RAxML and evaluated and optimized likelihood of the final tree under GAMMA (*10*). We created the phylogenetic tree in R 3.4.3 by using the ggtree R package (*11*). The tree was rooted on isolate GCA_003076555, the earliest isolate in the collection.

To determine the location of resistance genes, we used SPAdes (http://cab.spbu.ru/software/spades) for assembly and mapped contigs to the NYC ST307, isolate KP1768, core chromosome and plasmids. This indicated that the IncA/C plasmid harbored bla_{KPC}, bla_{TEM}, *sulI*, *aadB*, *aac6*, as well as *qacE*, implicated in chlorhexidine resistance. bla_{OXA-1}, $bla_{CTX-M-15}$ and bla_{SHV100}, as well as *catB*, *fosA*, *tet*, *aac6*, and *aadB* mapped to the ColR replicon, putatively integrated into the core chromosome. No major resistance genes mapped to the IncFIB(K) plasmid, which contained many elements for encoding resistance to diverse metals.

References

1. Clinical and Laboratory Standards Institute. Performance standards for antimicrobial susceptibility testing. 25th informational supplement. CLSI document M100-S25. Wayne (PA): The Institute; 2015.

2. Inouye M, Dashnow H, Raven LA, Schultz MB, Pope BJ, Tomita T, et al. SRST2: Rapid genomic surveillance for public health and hospital microbiology labs. Genome Med. 2014;6:90. PubMed https://doi.org/10.1186/s13073-014-0090-6

3. Cerqueira GC, Earl AM, Ernst CM, Grad YH, Dekker JP, Feldgarden M, et al. Multi-institute analysis of carbapenem resistance reveals remarkable diversity, unexplained mechanisms, and limited clonal outbreaks. Proc Natl Acad Sci U S A. 2017;114:1135–40. PubMed https://doi.org/10.1073/pnas.1616248114

4. Long SW, Olsen RJ, Eagar TN, Beres SB, Zhao P, Davis JJ, et al. Population genomic analysis of 1,777 extended-spectrum beta-lactamase-producing *Klebsiella pneumoniae* isolates, Houston, Texas: unexpected abundance of clonal group 307. MBio. 2017;8:e00489–17. PubMed https://doi.org/10.1128/mBio.00489-17

5. Wyres KL, Hawkey J, Hetland MAK, Fostervold A, Wick RR, Judd LM, et al. Emergence and rapid global dissemination of CTX-M-15-associated *Klebsiella pneumoniae* strain ST307. 2018 [cited 2018 August 11]. https://www.biorxiv.org/content/early/2018/06/21/352740

6. Seemann T. Snippy: Rapid haploid variant calling and core SNP phylogeny. 2017 [cited 2018 July 23]. https://github.com/tseemann/snippy

7. Arndt D, Grant JR, Marcu A, Sajed T, Pon A, Liang Y, et al. PHASTER: a better, faster version of the PHAST phage search tool. Nucleic Acids Res. 2016;44(W1):W16–21. PubMed https://doi.org/10.1093/nar/gkw387

8. Dhillon BK, Laird MR, Shay JA, Winsor GL, Lo R, Nizam F, et al. IslandViewer 3: more flexible, interactive genomic island discovery, visualization and analysis. Nucleic Acids Res. 2015;43(W1):W104–8. PubMed https://doi.org/10.1093/nar/gkv401

9. Stamatakis A. RAxML version 8: a tool for phylogenetic analysis and post-analysis of large phylogenies. Bioinformatics. 2014;30:1312–3. PubMed https://doi.org/10.1093/bioinformatics/btu033

10. Stamatakis A, Hoover P, Rougemont J. A rapid bootstrap algorithm for the RAxML Web servers. Syst Biol. 2008;57:758–71. PubMed https://doi.org/10.1080/10635150802429642

11. Yu G, Smith DK, Zhu H, Guan Y, Lam TTY. ggtree: an R package for visualization and annotation of phylogenetic trees with their covariates and other associated data. Methods in Ecology and Evolution. 2017;8:28–36. https://doi.org/10.1111/2041-210X.12628

12. European Committee on Antimicrobial Susceptibility Testing. Breakpoint tables for interpretation of MICs and zone diameters. Version 8.1, 2018. [cited 2017 Oct 10] http://www.eucast.org

Table 1. Results of antimicrobial susceptibility testing and molecular mechanisms of resistance of *Klebsiella pneumoniae* in patient in Dominican Republic with recent travel to Puerto Rico*

Antimicrobial drug	Mean inhibitory concentration (µg/mL) and EUCAST interpretation	Molecular mechanism
Meropenem	≥32 R	bla_{KPC-2}
Ceftriaxone	≥64 R	$bla_{CTX-M-15}$, $bla_{SHV-100}$, bla_{OXA-1}, bla_{TEM-1D}
Piperacillin/Tazobactam	≥64 R	
Cefepime	≥64 R	
Ciprofloxacin	≥4 R	*gyrA* Y83I, *parC* S80I
Gentamicin	≥16 R	*aac(3)-Iia, aac(6')-Ib, aac(6')-33, aadB*
Tobramicin	≥16 R	
Amikacin	16 I	
Colistin	<0.5 S	
Polymyxin	1 S	
Fosfomycin	NA R	*fosA3*
Trimethoprim/Sulfamethoxazole	≥320 R	*dfrA14, sulI, sulII*
Tetracycline	≥16 R	*tetD*
Minocycline	≥16 R	
Tigecycline	≥8 R	
Chloramphenicol	32 R	*catB4*

*Susceptibility testing for fosfomycin was performed by using disc diffusion testing; the zone diameter was 19 mm. Polymyxin B susceptibility testing was performed by using broth microdilution and meropenem testing was performed by using Etest (bioMérieux, https://www.biomerieux.com). Clinical and Laboratory Standards Institute breakpoints are not available for intravenous fosfomycin, however EUCAST criteria interpret the isolate as resistant (*12*). EUCAST, European Committee on Antimicrobial Susceptibility Testing; I, intermediate; NA, not available; R, resistant; S, susceptible.

Table 2. ST307 reference genomes from GenBank and metadata for previously published sequences used for comparative analyses of *Klebsiella pneumoniae* in patient in Dominican Republic with recent travel to Puerto Rico*

Isolate	Accession number	Location	Year	bla_{KPC}	bla_{CTX-M}
35111	SRR6892777	U.S. (New York, NY)	2014		CTX-M-15
35123	SRR6892773	U.S. (New York, NY)	2014		CTX-M-15
35476	SRR6892718	U.S. (New York, NY)	2015		CTX-M-15
35438A	SRR6892699	U.S. (New York, NY)	2015	KPC-2	CTX-M-15
ERR1218732	ERR1218732	Thailand	2015		CTX-M-15
ERR1218738	ERR1218738	Thailand	2015		CTX-M-15
ERR257620	ERR257620	UK	2010		CTX-M-15
ERR2631531	ERR2631531	Norway	2013		CTX-M-15
ERR2631532	ERR2631532	Norway	2012		CTX-M-15
ERR311471	ERR311471	UK	2012		CTX-M-15
ERR349773	ERR349773	Nepal	2012		CTX-M-15
ERR349787	ERR349787	Nepal	2012		CTX-M-15
GCA_001566595	GCA_001566595	Italy	2014	KPC-3	CTX-M-15
GCA_002166915	GCA_002166915	Colombia	2013		CTX-M-15
GCA_002166955	GCA_002166955	Italy	2014	KPC-3	
GCA_002167025	GCA_002167025	UK	2015	KPC-2	CTX-M-15
GCA_003076555	GCA_003076555	Iran	2009		CTX-M-15
KP1766	SRR6844958	U.S. (New York, NY)	2016	KPC-2	CTX-M-15
KP1767	SRR6844959	U.S. (New York, NY)	2016	KPC-2	CTX-M-15
KP1768	SRR6845005	U.S. (New York, NY)	2016	KPC-2	CTX-M-15
KP1769	SRR6845004	U.S. (New York, NY)	2016	KPC-2	CTX-M-15
NR0970	SRR9309433	U.S. (New York, NY)	2014	KPC-3	
NR5632	SRR6348596	U.S. (New York, NY)	2016	KPC-2	CTX-M-15
NR5706	SRR6348592	U.S. (New York, NY)	2016	KPC-2	CTX-M-15
**NR6025**	**SRR9309434**	**Dominican Republic**	**2015**	**KPC-2**	**CTX-M-15**
Reference	GCA_002148835.1	U.S. (Houston, TX)	2011	KPC-2	CTX-M-15
SRR5387157	SRR5387157	U.S. (Houston, TX)	2015	KPC-2	CTX-M-15
SRR5387161	SRR5387161	U.S. (Houston, TX)	2015	KPC-2	CTX-M-15
SRR5387164	SRR5387164	U.S. (Houston, TX)	2015	KPC-2	CTX-M-15
SRR5387169	SRR5387169	U.S. (Houston, TX)	2015	KPC-2	CTX-M-15
SRR5387172	SRR5387172	U.S. (Houston, TX)	2015		CTX-M-15
SRR5877450	SRR5877450	Cambodia	2013		CTX-M-15
SRR7345600	SRR7345600	Australia	2013		CTX-M-15
SRR7345601	SRR7345601	Australia	2013		CTX-M-15
SRR7345602	SRR7345602	Australia	2014		CTX-M-15
SRR851036	SRR851036	U.S. (Boston, MA)	2012		CTX-M-15

*Bold text indicates isolate in this case.
